# Demonstrating Functional Equivalence of Pilot and Production Scale Freeze-Drying of BCG

**DOI:** 10.1371/journal.pone.0151239

**Published:** 2016-03-16

**Authors:** R. ten Have, K. Reubsaet, P. van Herpen, G. Kersten, J.-P. Amorij

**Affiliations:** 1 Intravacc, P.O. Box 450, 3720 AL Bilthoven, The Netherlands; 2 Leiden Academic Center for Drug Research, Drug Delivery Technology, P.O. Box 9502, 2300 RA Leiden, The Netherlands; University College Cork, IRELAND

## Abstract

Process analytical technology (PAT)-tools were used to monitor freeze-drying of Bacille Calmette-Guérin (BCG) at pilot and production scale. Among the evaluated PAT-tools, there is the novel use of the vacuum valve open/close frequency for determining the endpoint of primary drying at production scale. The duration of primary drying, the BCG survival rate, and the residual moisture content (RMC) were evaluated using two different freeze-drying protocols and were found to be independent of the freeze-dryer scale evidencing functional equivalence. The absence of an effect of the freeze-dryer scale on the process underlines the feasibility of the pilot scale freeze-dryer for further BCG freeze-drying process optimization which may be carried out using a medium without BCG.

## Introduction

Lyophilization or freeze-drying is often used for stabilization of biopharmaceuticals such as vaccines [1, 2]. Besides improvement of the stability of vaccines, lyophilization is used to facilitate the production of new dosage forms of vaccines [3, 4], such as bioneedles for intramuscular delivery [5–8] or powders for pulmonary delivery [9, 10]. If properly formulated, lyophilized vaccines are less prone to chemical and physical degradation pathways owing to the removal of water and vaccine antigen vitrification in the formulation [11].

Bacille Calmette-Guérin (BCG) vaccine contains a non-infectious strain of *Mycobacterium bovis* and is used prophylactic against tuberculosis or for immune therapy against bladder cancer [1, 12–14].

The process of freeze-drying includes besides a freezing step also two drying steps: primary drying, and secondary drying. During primary drying ice is removed by sublimation and during secondary drying water is removed by desorption [2, 11, 15].

The strategy for the design of a freeze-drying process is generally based on the physical properties of the formulation in order to aim at a freeze-dried product with an intact cake structure. The occurrence of cake collapse, although not necessarily detrimental for the product, is often unwanted and may pose a reason for rejection of that vial. Collapse may occur during lyophilization by raising the product temperature (T_p_) above a critical threshold, the onset collapse temperature (T_oc_). The T_oc_ depends on the composition of the formulation and may be determined by freeze-drying microscopy (FDM) [15]. For amorphous solids, collapse may occur upon raising the product temperature to or slightly beyond the glass transition temperature of the maximally freeze-concentrated fraction (T_g_^ꞌ^). In the case of crystalline excipients, collapse may occur upon increasing the T_p_ to the eutectic temperature (T_e_). Both amorphous and crystalline components may be present in a single formulation [2].

Collapse can be prevented by choosing an appropriate shelf temperature and chamber pressure to ensure the T_p_ to remain below the T_oc_ during primary drying [2, 15]. Optimal process conditions for primary drying may be characterized by a minimum duration of primary drying and a maintained cake structure. This is relevant since freeze-drying is a lengthy process [2] especially in the case of non-optimized lyophilization cycles. In general, optimization of the duration of primary drying gives the biggest efficiency gain in lyophilization, resulting in less process time, and increased production capacity.

The aim of this study is to demonstrate product (RMC and BCG survival rate) and process (read: primary drying time) equivalence of pilot scale (total shelf area 2.7 m^2^) and production scale (total shelf area 43.2 m^2^) freeze-drying of BCG to support further BCG freeze-drying process optimization at pilot scale. This will be done by using both well-known PAT-tools, the pressure rise test (PRT) [16], T_p_ [17], a balance [18], and the condenser temperature [19], and a, to our knowledge, novel PAT-tool, which is the open/close frequency of the vacuum valve used for chamber pressure control. This frequency is indirectly determined from the saw-tooth pattern in the recorded graphs of the registered chamber pressure during freeze-drying. Process equivalence may also be demonstrated by showing that T_p_-profiles (T_p_ vs time) are independent of the freeze-dryer scale [20–23], however, GMP-restrictions, did not allow measurement of the T_p_ at production scale. For that reason, alternative in-process parameters were considered as (scale-dependent) PAT-tools to monitor primary drying and determine the endpoint thereof.

The comparison of freeze-drying processes as a function of dryer scale has been documented before [24, 25]. This study contributes to the existing knowledge by providing a substantial experimental data set on two freeze-drying cycles, which gives insight in process variation and equipment related factors (if present).

## Materials and Methods

### Description of formulation, products and materials

After cultivation, BCG was pelleted by centrifugation and formulated in freeze-drying medium [14, 26]. One liter of freeze-drying medium (HGT-medium) contains 108 g glucose, 25 g polygeline (Thera Select unless stated otherwise.) and 0.05 g Tween80.

Prior to freeze-drying 23 mL vials are filled with either MiliQ (purified water), HGT-medium, or formulated BCG to reach a total volume of 10 mL/vial (fill height = 2.0 cm) which are equipped with stoppers (Helvoet Pharma, The Netherlands).

Freeze-drying was performed at pilot scale (Klee, shelf surface area: 2.7 m^2^) and/or production scale (Klee, shelf surface area: 43.2 m^2^). The pilot freeze-dryer contains 5 shelves, including a sampling shelf and balance shelf. The sampling shelf is suitable to be completely transported to the interior of a transparent Perspex chamber mounted on the freeze-dryer door from where vials can be closed and/or removed during the freeze-drying process using a sample thief.

### Freeze-drying protocols

Freeze-drying was performed according to two cycles, I and II (see Table 1).

**Table 1:** Freeze-drying protocols as used in this study (PD = primary drying, and SD = secondary drying). The pressure rise test (PRT) in cycle I was used to confirm the end of primary drying. In cycle II the PRT was used during primary drying and after not meeting the criterion (maximum rise in pressure <0.03 mbar/min) the primary drying stage was prolonged by an additional 2 hours.

	Cycle I			Cycle II		
Step	t (hours)	T (°C)	p (mbar)	t (hours)	T (°C)	p (mbar)
Loading		+4	~1000		+4	~1000
Equilibration	3	to -15	~1000	3	to -15	~1000
	1	-15	~1000	1	-15	~1000
Freezing	1	to -35	~1000	1	to -35	~1000
	1	-35	~1000	1	-35	~1000
PD	1	-35	0.045	1	-35	0.09
	0.5	to -30	0.045	0.5	to -30	0.09
	227, PRT	-30	0.045	90	-30	0.09
				2, PRT	-30	0.09
SD	30	+30	0.045	30	+30	0.09
	30	+30	0.007	30	+30	0.046

In these cycles, a PRT was performed as indicated. If the PRT-criterion (pressure rise < 0.03 mbar/min) of cycle II was not met, an extra drying time of 2 hours was added after which another PRT was performed ultimately till the end of primary drying was reached (the PRT-criterion was met) after which secondary drying was started.

The difference in chamber pressure during secondary drying between cycle I and II was presumed not to impact product quality since the water desorption rate in the cycles used is independed of the chamber pressure below 0.27 mbar [2].

### Demonstrating equivalent drying behavior of HGT-medium and BCG in HGT-medium

Drying of HGT-medium and BCG in HGT-medium was investigated using a fully loaded pilot freeze-dryer. The freeze-dryer predominantly contained BCG in HGT-medium. On the sampling shelf, HGT-medium and BCG in HGT-medium were placed next to each other (in sets of three each). A sample thief was used to stopper and remove vials during freeze-drying according to cycle II.

### Effect of chamber pressure on the sublimation rate

A series of independent experiments was performed in the pilot freeze-dryer to study the effect of the chamber pressure (from 0.01 to 0.5 mbar) on the sublimation rate. Vials were filled with either 10 mL MiliQ or HGT-medium, stoppered, and loaded onto the balance shelf (~ 79 vials) and the sampling shelf (~ 79 vials). In order to reduce the impact of external heat radiation, the Perspex chamber was covered with aluminum foil. Freezing was performed as described before (see Table 1). During primary drying at the studied chamber pressure, the shelves were held 1 hour at -35°C, then in 0.5 hour to -30°C and thereafter, up to 40 hours at -30°C. The average sublimation rate to remove 100 g of ice was calculated from the decrease in mass over time.

### Analytical testing

#### BCG survival

For determination of the BCG survival, a dilution series was prepared in Sauton solution (freeze-dried material was resuspended in physiological salt solution) and the number of viable BCG bacteria, before and after freeze-drying, were determined by counting the colonies grown (at a temperature of 36 ± 0.5°C for at least 28 days) on Loewenstein agar plates.

Results are reported as the average BCG survival of three randomly selected vials (this represents an analysis result).

#### Residual moisture content

The residual moisture content (RMC) was determined using a Karl Fischer coulometric titrimeter (Model CA-06 moisture meter, Mitsubishi). The samples were weighed, subsequently reconstituted in 10 mL Coulomat A (Fluka, Switzerland) and a volume of 0.1 mL was injected into the titration vessel. The mass of the dried material was calculated by also determining the mass of the empty vial (+stopper). The RMC was calculated based on the measured water content, the mass of the dried material, the reconstitution volume, the injection volume and the water content of the blank.

To obtain an analysis result, the average RMC of three vials (randomly selected from the batch) was calculated.

### Statistical analysis

The statistical analysis were performed using Student’s t-test with P < 0.05 as the minimal level of significance.

The number of experiments (n) refers to the number of analysis results, or primary drying times from independent experiments.

Average values are presented ± standard deviation.

## Results

### Scale-dependent PAT-tools for monitoring primary drying

Several in-process parameters, PAT-tools, are available for monitoring BCG primary drying at pilot and production scale (see Table 2) of which the PRT is available in both freeze-dryers.

**Table 2:** Overview of in-process parameters at pilot and production scale that are useful for monitoring primary drying and primary drying endpoint determination.

	Feasibility for primary drying endpoint determination	
Parameter	Production scale	Pilot scale
Pressure rise test	+	+
Product temperature (T_p_)	-	+
Mass (balance shelf)	-	+
Outlet condenser temperature	+	-
Vacuum valve*	+	-

* Refers to changes in the chamber pressure caused by opening and closing of the vacuum valve.

**Fig 1:**
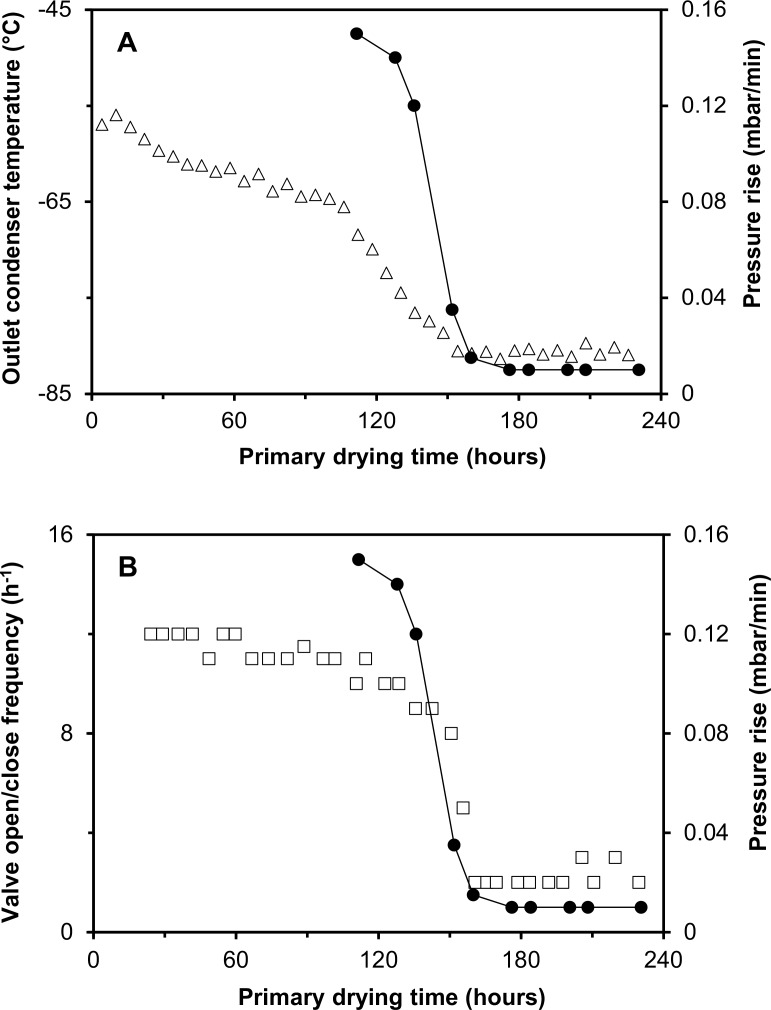
Measured in-process parameters during freeze-drying of BCG at production scale. Panel A shows the outlet condenser temperature (clear triangle). Panel B shows the open/close frequency of the valve connecting the chamber and the vacuum pump (clear square). In both panels the rise in pressure in one minute from the PRT is shown (black circle).

At production scale, the PRT is only routinely used to confirm the endpoint after the fixed primary drying time of 228.5 hours. By using the PRT for process monitoring (see Fig 1) it can be seen that this fixed period of time is longer than strictly required, *i*.*e*. the required time for primary drying is 150–160 hours as by then the pressure rise is very low and almost constant.

The observed trend in values from the PRT corresponds to time profiles of the outlet condenser temperature (see Fig 1A) and the vacuum valve open/close frequency (see Fig 1B) evidencing the use of these methods for monitoring the primary drying process.

In the pilot freeze-dryer, use of the balance shelf and external thermocouples were evaluated for determination of the endpoint of primary drying (data not shown). It appeared that passing the PRT criterion also coincided with: A) no further decrease in mass of vials placed on the balance shelf and B) a constant apparent product temperature that is generally above the shelf temperature.

### Functional equivalence of BCG freeze-drying

#### Freeze-drying according to cycle I

Production scale freeze-drying of BCG is done according to a non-optimized freeze-drying cycle (cycle I) which consistently yields product (see Table 3) with a BCG survival rate of 7.4 ± 2.5% (n = 14), and an RMC of 0.78 ± 0.08% (n = 14).

**Table 3:** Overview of primary time (hours), RMC (%), and BCG survival rate (%) as a function of the freeze-drying cycle and freeze-dryer scale. Several methods were used for primary drying endpoint determination: the vacuum valve open/close frequency, the outlet condenser temperature, and PRT refers to the pressure rise test. The maximum load of the freeze-dryers is as follows: 12500 vials at production scale and 750 vials at pilot scale. See S1 Data for raw data.

			Final product	
Description	Vials	PD time (hours)	RMC (%)	Survival (%)
**Cycle I**				
*Production scale*				
Fixed time	~ 10500	228.5	0.78 ± 0.08 (n = 14)	7.4 ± 2.5 (n = 14)
Vacuum valve		164.2 ± 3.1 (n = 6)		
Outlet cond. temp.		158.3 ± 5.7 (n = 6)		
Overall		161.3 ± 6.5		
*Pilot scale*				
• PRT	~ 750	153.5 (n = 2)	0.82 (n = 2)	-
	~ 160	150.7 (n = 1)	-	-
Overall		152.5 ± 5.5		
Cycle II*				
*Production scale*				
• PRT	~ 10500	129.0 ± 2 (n = 3)	0.83 ± 0.06 (n = 3)	8.4 ± 2.5 (n = 3)
*Pilot scale*				
• PRT	~ 750	120.6 ± 6 (n = 4)	0.80 ± 0.07 (n = 3)	7.7 ± 3.0 (n = 3)
	~ 160	131.3 (n = 1)	-	-
Overall		122.9 ± 7.0		

* The supplier of the polygeline component was Piramal.

Estimating the duration of primary drying using the vacuum-valve-method and the outlet-condenser-temperature-method indicated that a duration of ~162 hours (instead of 228.5 hours) appeared already sufficient to remove all ice. Pilot scale freeze-drying resulted in an average primary drying time of ~153 hours, which was not significantly different from the production scale value (p > 0.05).

#### Selecting a more favorable chamber pressure

**Fig 2:**
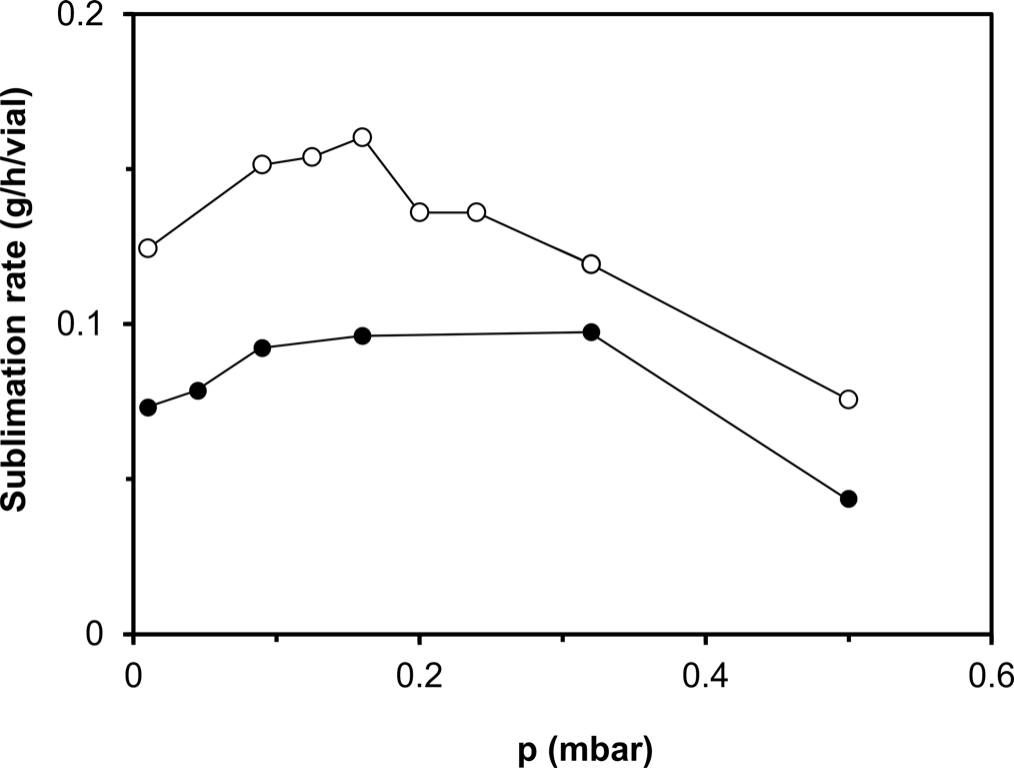
Effect of the chamber pressure on the average sublimation rate of HGT-medium (black circle), and purified water (clear circle) in the pilot freeze-dryer. The sublimation rate was calculated from the time course to sublimate 100 g of ice.

In order to study the effect of a process change as function of freeze-dryer scale, a more favorable chamber pressure was selected by confirming the well-known effect of the chamber pressure on the sublimation rate [25, 27]. Fig 2 shows that a higher sublimation rate may be obtained by applying a chamber pressure of 0.09 mbar (in cycle II) instead of 0.045 mbar (as used in cycle I) to freeze-dry both purified water and HGT-medium. In all cases, the HGT cake structure was maintained during the course of the experiment.

#### Freeze-drying according to cycle II

At production scale, a primary drying time of ~129 hours was obtained, a gain in process time of ~30 hours compared to cycle I (see Table 3). The BCG survival rate was 8.4 ± 2.5% and the RMC 0.83 ± 0.06%. The primary drying time, BCG survival rate, and RMC did not differ significantly (p > 0.05) from those observed at pilot scale. The results from cycle I and II were used to demonstrate functional equivalence of pilot and production scale freeze-drying of BCG.

The feasibility of the pilot scale freeze-dryer as a down-scale model for further freeze-drying cycle optimization was confirmed further by freeze-drying an experimental HGT-medium (without BCG).

**Fig 3:**
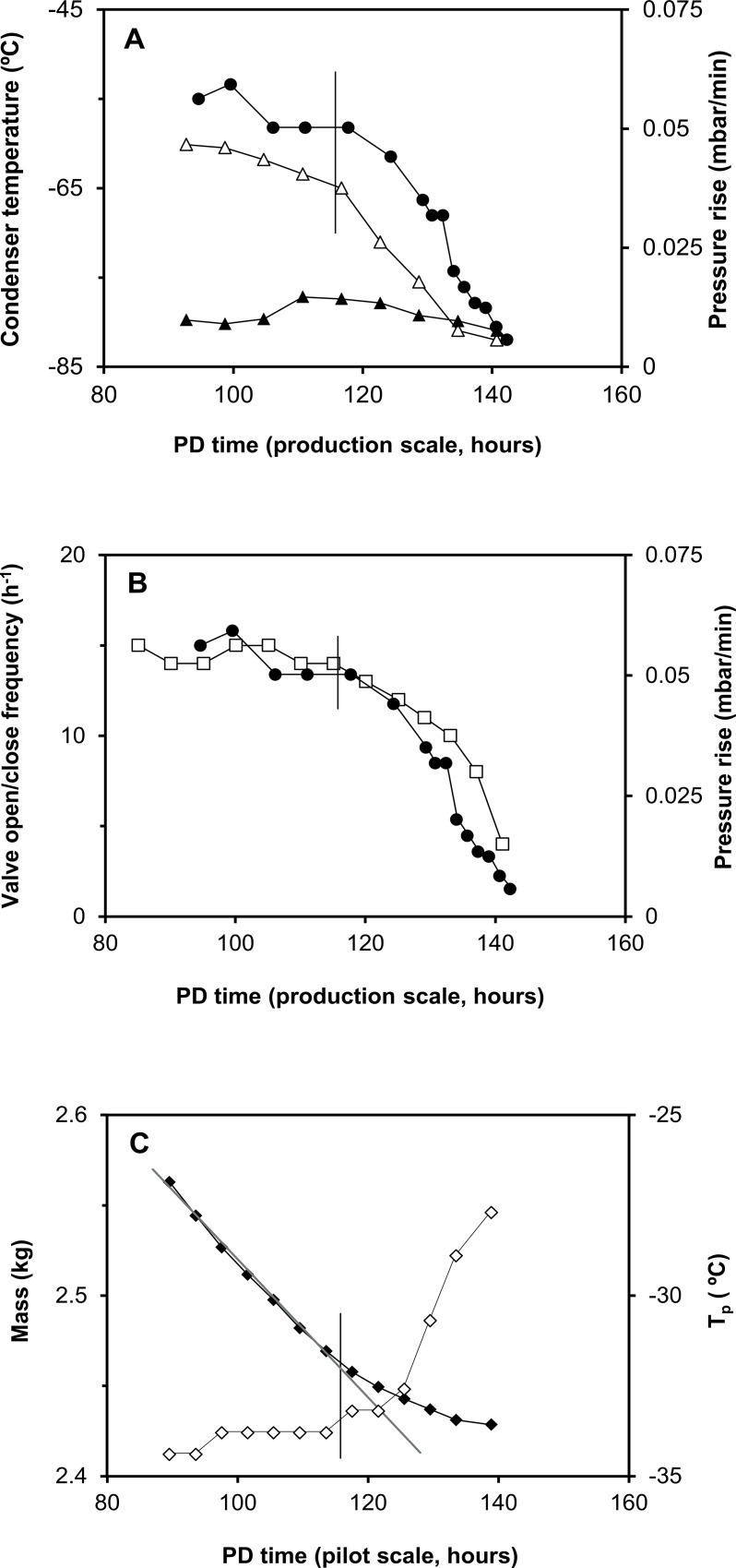
A comparison of in-process data upon using the shortened freeze-drying process during primary drying (PD) of HGT-medium (supplier of the polygeline was BBraun) at production scale (panel A and B) and pilot scale (panel C) in the time-frame from 90 hours till the end. Panel A shows the outlet (clear triangle) and inlet condenser temperature (black triangle), and the pressure rise obtained after 1 minute performing the PRT (black circle). Panel B shows the open/close frequency of the valve between the vacuum pump and the chamber (clear square). The pressure rise from the PRT is included as well for comparison (black circle). Panel C shows the decrease in mass (black diamond) and the temperature of a vial that is placed in the centre of the array and thereby shielded from outside heat radiation (clear diamond). The straight grey line in panel C serves as a visual aid to show a reduced decrease in mass beyond ~116 hours. The vertical line in panel A-C marks a primary drying time of ~116 hours after which marked changes in the studied in-process parameters are noted.

The available and relevant in-process parameters showed an evident change after ~116 hours of primary drying (see the vertical line in Fig 3A–3C).

This change likely illustrated a decrease in the overall sublimation rate, as concluded from the slower loss in mass of vials on the balance shelf in the pilot scale freeze-dryer beyond ~116 hours of primary drying (see Fig 3C). This time course may be explained by a decreasing number of vials still containing ice, *i*.*e*. the drying rate of edge vials is known to be higher than that of centre vials [24, 28–30]. Therefore, the vertical line in Fig 3A–3C apparently marks the moment at which vials at (and/or close to) the edge no longer contain ice. This suggests that primary drying of edge vials was independent of the dryer scale.

At production scale, the inlet condenser temperature (see Fig 3A) showed a constant minimum of ~-80°C. Apparently, there is a substantial amount of heat transferred to the condenser during solidification of water vapour onto the condenser, which is reflected by a higher initial outlet condenser temperature of ~-60°C. A lower overall sublimation rate (beyond ~116 hours) results in a lower outlet condenser temperature because less heat is transferred to the condenser. This means that the observed pattern in condenser outlet temperature is related to the cooling capacity of the equipment.

The observed pattern in the vacuum valve open/close frequency of the production scale freeze-dryer (see Fig 3B) may be explained by the decreasing need to adjust the chamber pressure near the end of primary drying.

The increase in pressure from the PRT was included as a control (see Fig 3A and 3B) and was found to parallel the outlet condenser temperature and the vacuum valve open/close frequency. This confirmed the suitability of both methods for primary drying endpoint determination as described earlier.

In the pilot scale freeze-dryer, the apparent product temperature of a centre vial on the sampling shelf is clearly below the shelf temperature of -30°C during sublimation (see Fig 3C). The product temperature increases as cooling of the vial decreases, beyond a primary drying time of ~116 hours. At the end of primary drying, the product reaches a temperature above the shelf temperature due to outside heat radiation.

**Fig 4:**
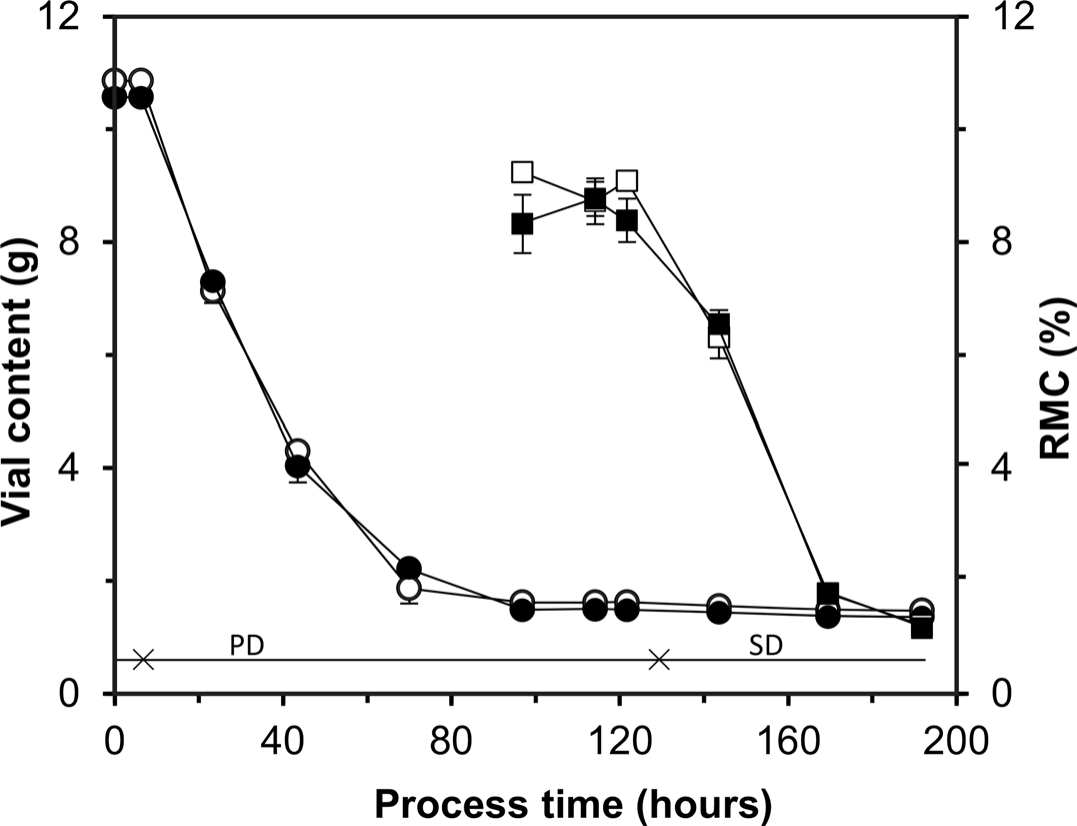
Freeze-drying of 10 mL HGT-medium and BCG in HGT-medium in the pilot freeze-dryer. Depicted are: the vial content (g) of BCG in HGT-medium (black circle) and HGT-medium (clear circle) and the RMC (%) of BCG in HGT-medium (black square) and HGT-medium (clear square). Each dot represents an average value ± standard deviation (n = 3). The solid horizontal line shows represent the total process time. The stars mark the beginning of primary, and secondary drying.

In an independent experiment, it was tested whether freeze-drying of HGT-medium would be representative for BCG freeze-drying. Fig 4 shows that the removal of moisture, reflected by a decrease in vial content and RMC, is comparable. This shows that further cycle improvement may be performed with HGT-medium (or mostly HGT-medium and some vials containing BCG).

## Discussion

### PAT-tools for monitoring primary drying and demonstrating process equivalence

This study introduces a novel PAT tool, the vacuum valve open/close frequency for monitoring primary drying and determination of the primary drying endpoint. Opening and closing of the vacuum valve resulted in a typical saw-tooth pattern in the chamber pressure (not shown) which was registered during BCG freeze-drying. The fact that the production scale process is apparently not operated at a constant chamber pressure is not ideal and for that reason, most production scale freeze-dryers are currently equipped with a calibrated leak. In such a case, it is possible to use the bleed rate of (sterile) N_2_ into the freeze-dryer as a PAT-tool for process monitoring [19].

Besides these two tools, there are many more available in the literature [16] of which use of the PRT [16], T_p_ [17], balance [18], and condenser temperature [19] as used in this study are well-known examples.

Demonstrating process equivalence is preferred in case of process transfer to another freeze-dryer. In such a case, the product temperature vs time profiles should ideally be the same [20–23]. With respect to the duration of primary drying, the T_p_ of centered vials is relevant as these are generally colder than edge vials and will need more time to dry. Edge vials may be relevant for studying the visual appearance of the cake. In this study, the visual appearance (mostly intact with only small dents at the bottom) of the cake was independent of the dryer scale and position of the vial indicating that in all cases the T_p_ was below the critical threshold and therefore visible cake collapse did not occur.

This study shows that besides measurement of T_p_ also other in-process parameters appear useful to demonstrate process equivalence upon operating different freeze-dryers as also explained elsewhere [19].

### Effect of freeze-dryer scale and other factors on primary drying

There are several examples described in the literature on the comparison of processes operated in different freeze-dryers [24, 25, 31]. Running the same cycle in different freeze-dryers is no guarantee for process equivalence as several (scale dependent) factors may affect the process [19–21].

A GMP production scale freeze-drying environment may be cleaner (less particles) than a research setting which in turn may result in a higher degree of supercooling during freezing at production scale. Compared to pilot scale freezing, this may result in the formation of smaller ice crystals, a higher average T_p_, and a longer primary drying time approximating 1% of the total PD time/°C of extra supercooling [17, 18]. In this study, no difference in primary drying time was noted. Probably, the equilibration step, in which the vials are kept at -15°C for 1 hour before freezing, and/or the presence of sufficient (bacterial) nucleation sites [32] minimize(s) freezing differences.

Also the dryer load may affect the primary drying time [22]. In this study, this possibility was addressed by lowering the number of vials in the pilot scale freeze-dryer. It was found that a lower load of 20% (a fully loaded middle shelf, i.e. loaded with two cassettes) did not result in a reduced primary drying time (see Table 3) likely because the percentage of warm edge vials remained unchanged.

Interestingly, the observed variation in the determined primary drying time (see Table 3) is relatively small (RSD < 6%). This experimental base is in support of a theoretical approach, which allows calculation of for example the effect of shelf temperature on the primary drying time. Typically, such calculations appear satisfactory [23], suggesting that inherent freeze-drying process variation is small in general.

### Outlook towards process time reduction of BCG freeze-drying

It was demonstrated here that pilot scale freeze-drying resembled the production scale process suggesting suitability of the pilot scale freeze-dryer for future process optimization. For optimization experiments, use of HGT-medium (for example supplemented with some BCG vials) is possible as drying was not affected by the presence of BCG (see Fig 4).

It is conceivable that process time reduction may be obtained by increasing the shelf temperature. A short study indicated that the use of a shelf temperature gradient during primary drying could significantly reduce the primary drying time without affecting the cake appearance (data not shown), but this cycle was not implemented at production scale.

Besides shortening the duration of primary drying it may also be possible to reduce the secondary drying time by for example increasing the shelf temperature ramp rate of the first secondary drying step (see Table 1) as explained elsewhere [2].

### Risks related to process transfer from a pilot to a production scale freeze-dryer

The freeze-drying cycles studied here use a constant shelf temperature for primary drying. It was anticipated that this would mitigate risks involved in process transfer, i.e. avoid process differences associated to uneven heating of shelves, and/or temperature lag of which both are conceivable in case of a (steep) shelf temperature gradient. The introduction of extra primary drying time (10 h) after the estimated endpoint of primary drying would mitigate the risk for the occurrence of melt back in vials located in the center of an array (not required in this study).

The risk for the occurrence of evident cake collapse in edge vials was not anticipated (at least in the case of BCG freeze-drying) because the impact of heat radiation is generally less in a production scale freeze-dryer.

## Supporting Information

S1 Data Raw data corresponding to Table 3. (XLSX)

## Abbreviations

PAT: Process analytical technology
BCG: Bacille Calmette-Guérin
RMC: Residual moisture content
cGMP: Current good manufacturing practices
FDM: Freeze-drying microscopy
T_p_: Product temperature
T_oc_: Onset collapse temperature (determined by FDM)
T_e_: Eutectic temperature
Tgꞌ: Glass transition temperature of the maximally freeze-concentrated fraction
PRT: Pressure rise test
